# The Gulstonian Lectures on Neuroses of the Viscera

**Published:** 1884-05

**Authors:** T. Clifford Allbutt

**Affiliations:** Lecturer on Practice of Physic at the Leeds School of Medicine; Consulting Physician to the Leeds Hospital for Women and Children


### THE GULSTONIAN LECTURES ON NEUROSES OF THE VISCERA.

Delivered at the Royal College of Physicians, March 14, 1884. By T. CLIFFORD ALLBUTT, M.A., M.D., F.R.C.P., Lecturer on Practice of Physic at the Leeds School of Medicine; Consulting Physician to the Leeds Hospital for Women and Children.

GENTLEMEN:—When the revered chief of this college bade me to the high place which I now hold, I would fain be released from so great a call. It seemed to me that the leisure in which I should search out and think things worthy of so distinguished an assembly had long been denied to me. But I was assured that, although lectures containing the results of systematic research are of the highest importance, there is a place, nevertheless, for lectures conveying the humbler reflections of working physicians who deal with the daily experience of the sick room and the consulting chamber. It remained for me, therefore, to obey, and to deal with some part of the field of practice. Herein I have chosen for my subject those painful disorders which have their seat in the nerves of the viscera, but I have entitled my lectures "The Neuroses of the Viscera," in order that I should be free to enlarge occasionally upon some perturbations of those nerves which are not attended with pain, strictly so called. At the same time, it is with such neuroses as are manifested in pain that I intend more especially to deal. It seems to me that in our text books these affections receive something less than the full treatment which their difficulty and importance demand, and I find, in general practice, that the part played by the nervous system in visceral disorders is insufficiently seen. To the knowledge and skill of physicians so highly placed as yourselves I can not hope to add anything, but I would rather seek, through you, and with your aid, to spread abroad a more adequate interpretation of the disorders in question.

It certainly happens that neuroses above the belt are far more clearly understood than those below. This preference is due in part to the renewed impulse given to the study of thoracic disease by the discoveries of Laennec, partly to the more vivid functions of these parts, functions which excite our admiration and interest in greater measure than the brooding and silent life of the organs of vegetative existence. Of the neuroses, then, of the upper chambers of the body, of migraine, of asthma, of angina pectoris and the like, I shall find but little to say; my attention will be confined almost entirely to the pains and storms of the abdominal regions—to gastralgia, nephralgia, hepatalgia and their allies. Of the neuroses of the pelvic viscera much has been said. Indeed, if thoracic neuroses have been discussed at their actual value, if those of the abdomen have received less than their due meed of attention, surely those of the pelvis have received an attention, if not beyond their importance, at any rate of far too exclusive a bearing. What I say of these last will be little in description, but more in the way of interpretation.

The first strong impression which I received from the facts of the class with which we now concern ourselves was obtained from the index of my case books. It has been always my custom to index therein the names not only of my patients, but also of their maladies. I have to each volume two indices, one of names, one of diagnoses.

As each year I have prepared the spaces for these tables, I have been struck with the small space occupied by the malady "dyspepsia." Surely, I thought, if there be one malady which above all others should need a large space it must be dyspepsia. Yet year by year, as at the end of them I cast my eye over the tables, was I astonished to find my cases entered as "dyspepsia" to be a mere handful. How could this be? I asked myself repeatedly. "Martyrs to dyspepsia" are to be found at every street corner, and are said to form something little less than the staple of those who drift from consultant to consultant. Moreover, I had some suspicion that I was in repute as a stomach-doctor, and was, at least, conscious of a readiness to receive such cases, and to work patiently with them. I thought also of that long suffering organ itself, and its work—of the greediness or recklessness which make it the helpless receptacle of all sorts of rubbish; I pictured to myself the contents of the viscus distended with greasy, acidulous, or trashy matters, sodden in half-fermented gooseberry-juice or sour claret; and I stood amazed that it resists destruction. Yet not only does it survive, but it patiently draws order out of disorder, sweetness out of foulness. Again, as I turned to the journals of the day, I read of men of lofty endowments, whose lives became accursed by dyspepsia; and on other sheets I read many columns of advertised solace, from wind-pills to the pill of the American doctor, whose virtues were thus rendered at Cambridge:

> "Hæc jecur instigat, stimulos hæc renibus addit,
> Ambulat arcanas hæc taciturna vias.
> Attamen hæc eadem latebras penetralibus imas
> Mordacis buxi more modoque petit."

Our medical journals all tell us the same story. Every large drug-house has its pepsines, its dinner-pills, its cordial bitters, testifying not only to the general, but to the medical, cry for help against the demon of dyspepsia. Finally, so confident is the public of the true form of its enemy, that one of this sort who seeks the doctor will dictate his diagnosis, declaring himself an old dyspeptic, and demanding your specific. A hundred doctors have pronounced him dyspeptic; needlessly, for who can know it better than himself! He will condescend, perhaps, to admit some discussion of the respective vices of his stomach and of his liver, but he is offended if you seek further. He half tells you that, if you have yet to learn what is meant by a dyspeptic or by "biliousness," you are no man for him. Patients have positively left me in dudgeon because I ventured to hint that the word indigestion expresses rather an inference than a fact, and pointed out that diagnosis belongs to the physician, and not to the client.

How is it, then? How are we to explain this catholic wailing over a disease which is not; this wealth of balsams for sufferings which cannot be named? Is there no distress to lull, no pain to lenify? In turning our minds to this question, let us, in the first instance, consider the meaning of the word dyspepsia, and ask ourselves if there be in it any such confusion as may account for its indefinite uses, and whether in such uses there be equivocations such as to account for the doubts to which I have referred. I believe that, if I thus follow the origin and paths of my own thoughts in the past, I shall best open out the main subject of my address. Forgive me if I thus approach the subject of visceral neurosis by an indirect method, and in the way of comment upon the affections of the stomach. It is almost trivial to remark that dyspepsia, strictly speaking, is not a disease, but a symptom; it names not the causes nor the processes of the evil, but its consequences; that, as an outcome of the affair, whatever it may be, food is not digested, or is digested imperfectly or painfully. Taken in this sense, no doubt dyspepsia is an exceedingly common complaint. But if the word be used in this sense, does not its meaning become almost futile? As a consequence of various conditions, let us say, persons may find themselves unable to walk far, to walk steadily, to walk painlessly, or even to walk at all. Yet are we to use a word such as dyskinesis, and to say that whosoever comes to us walking ill, whether from general debility, from palsy, from sciatica, from muscular rheumatism, from gout, from disease of foot, ankle, or knee, that he is suffering from dyskinesis? Are we to say, of a patient whose forces are so diminished that he can only walk a mile, and this with fatigue, that he is suffering from atonic dyskinesis? Are we to treat all the above maladies in a chapter under the heading of dyskinesis, and in a few columns of entanglement try to deal intelligibly with such a subject in such a way? We should not be saved from the discredit of foolishness if we were to do so. Then, how are those to be spared who, using the word dyspepsia in this way, devote chapters and books of chapters to such a symptom, and raise that which is accidental and consequent to the place and name of a definite malady? Yet I have only to turn to any modern text-book to find page after page of such writing, pages to which I would willingly refer accurately, were it not that I would avoid even the seeming of personal controversy with authors whose industry and thoughtfulness have done so great a work for modern medicine. If, however, we would speak wisely and strictly, shall we not seek to clear away this kind of confusion, and set out in their natural groups, as distinctly as we can, the several classes of symptoms now heaped together unreasonably? Thus we shall better understand the cases which come before us, and taking a larger view of them, see better how to cure them. Perhaps, too, by this method, we shall even do away entirely with the word dyspepsia as the name of a malady, and reduce it to the position, for instance, of the word cough, which has no definite connotations, and signifies only an inconvenient and notorious symptom common to many maladies.

Sifting, then, as we must, the heap of affections attended with dyspepsia, we readily enough propose four chief groups of stomach disorders. First, those which are due to grave disease of the stomach itself, such as cancer or ulcer of the organ, dilatations of it, atrophy of its coats, and so forth. Secondly, to diseases of the stomach less grave, but still local, such as the simpler catarrhs, acute and chronic gastritis. Thirdly, disorders which depend upon no visible changes in the structure of the stomach, but consist in some disorder of its work or secretions, and which, in accordance with a convenient use of words, we call functional; the dyspepsia of gout may perhaps be taken as an instance of this group. Fourthly, disorders which depend, not upon any primary derangement of the tissues of the stomach, but upon some influence coming from blood or nerve, from which influence visible local changes may or may not ensue. We put aside the first class of cases, without difficulty, as quite foreign to our purpose; and, so far as they can be distinguished, we put aside the second class also. This it is easy to do in the instance, say, of chronic or acute gastritis caused by the excessive use of alcohol; or, again, in the instance of that common and transient catarrh of the stomach attended with loss of appetite, headache, coated tongue, constipation, sickliness and malaise, which is due often to cold, often to improper feeding. This affection accounts for a large number of the cases called bilious attacks, though the majority so called are probably migraine. It is easy to make some such progress as this, but thenceforth it is not easy to plan out our schedules further. We may leap over a score of doubtful cases, and passing at once to another extreme, separate into a class all cases which are obviously neuralgic; but between these positions lie a great number of cases which are hard to classify. Is pyrosis, for instance, some local affection of the coats of the stomach, or is it a neurosis? Is flatulence, are all acid risings in the stomach, due to some irregular transmutation of the contents of the organ, or are some of them consistent with the course of normal, if feeble, digestion? In the former case, such derangements may fairly be called dyspepsia, for the process of digestion goes wrong, not as a detail or subordinate part of larger or systemic irregularities, but as a simple and local error in the digestive work of the stomach itself. In a word, is stomach derangement, in a given case, but an expression of some general derangement, or is it a substantial ailment, and the cause rather than the consequence of any more general perturbations. Even these may be very difficult to estimate, or may be decided only on the study of individual cases, but a large number of cases remain which are yet even harder to interpret, cases in which dyspeptic symptoms are no doubt due to disordered work or secretion in the stomach, but in which this disorder of work or secretion may, in its turn, be due, not to any primary defect in that organ, but to some cause lying outside of its peculiar tissues, lying, for instance, in the blood or in the nervous system. Omitting, as I must do, all reference to blood changes, let us take as an extreme instance a sudden nervous shock, which may arrest digestion completely, and we may thereafter conceive of lesser degrees of nervous perturbation, which may bring up a continued interference with digestion, or, in other words, cause a continual dyspepsia. We may conceive, indeed, that not only in this way digestion may simply be slowed, but also the peptic secretions or processes be positively vitiated, as is the case, say, in neurotic diarrhœa.

Or, to turn to a different point of view, a patient who suffers from many symptoms indicative of nervous derangement tells you that on the empty stomach—generally in the morning, before breakfast—a dense, yellow, oily fluid gathers, and, lying there all day, would vitiate the viscus, would act as an eccentric cause of headache, and as a foul ferment upon the food in the course of digestion; so that his or her only hope of a comfortable day is to vomit or wash out these dregs at the beginning. Now, such an exudation may be due to some chronic distemper of the coats or glands of the stomach; but I incline to believe it is due rather to some perturbed innervation of a stomach otherwise healthy, seeing that the symptom is one which I have always found in neurotics, and to be curable only by treatment planned mainly upon this diagnosis. Disordered work and distempered secretions, then, may well be due, and doubtless often are due to neuroses of the stomach; and such neuroses, lying between the more localized disorders and the purer neuralgias, are difficult to classify. There is one more difficulty, though in practice a less embarrassing one; namely: that, as distempered secretion may be the effect of disordered nerve, so, reversely, some slighter catarrh, or other local change, or some offending article of diet, or, again, some graver local mischief, may, as peripheral irritants, set up nerve disturbances, which, in certain susceptible persons, may so wax as to overshadow or conceal the primary cause of their occurrence. We know, for instance, that the touch of a bronchial attack, or of an acute pneumonia, may first reveal an asthma till that moment wholly latent—latent, it may be, till middle or even later life; or latent it might have been, like the unwept tear, forever. But the sleeping ill, once awakened, rarely recedes again altogether, but, by its own recurrence, tends to rivet upon the sufferer the chains of habit.

Thus it may become difficult to say which is the predominant factor in the consequent group of discomforts. As a practical difficulty, it is most serious in cases in which ulcer may or may not be present; and I do not hesitate to say, gentlemen, even before you, that in some of these the diagnosis between ulcer and pure gastralgia is, in certain stages, impossible. How are we, then, to succeed as ministers to the sick if we crowd into one chapter, and almost into one point of view,the pure neuralgias of the stomach, the neuralgias awakened by local irritations within the viscus, disordered secretions or metabolisms within it due to perturbed innervation, together with primary dyspepsias of local origin which concern the nervous system but little or not at all? Never can we succeed, I think, if we make such a confusion. We must endeavor,then,not only to separate the pure neuralgias from the dyspepsias or pains of local origin; but, however difficult it may be in any one instance, we must endeavor farther to decide concerning mixed cases, whether the neuralgiac phenomena stand in causal relation to the local disorders of function, or contrariwise, the disorders of local function have awakened nervous reverberations. That which reason and examination may fail to discern may often be revealed to us by the test of treatment. The relation of asthma to disorders of the lungs has been quoted as an illustration of these difficulties. In some cases, then, we have to deal with a pure neurosis of central origin; in others, with nervous phenomena awakened by persisting or foregone local maladies; and unless we see with some clearness how these phenomena are related to each other, we shall fall short of a rational therapy.

In order to enter by the plainest route into the more intimate knowledge of neuroses of the stomach, let us advance from the simplest to the more complex. No cases, perhaps, will serve us better as an introduction than those in which disorders of digestion occur as a consequenee of general nervous exhaustion. Unlike gastralgia, these disorders may arise in men and women of very various habit of body. No general sketch of the bodily aspect, nor of the temperament of such patients, can be delineated, and accordingly we find the symptoms various.

Omitting,then,persons disposed to gastralgia, we find two kinds of dyspepsia at least arising in persons overworked. In the first class, we find simply a feeble stomach, as we find enfeebled legs and enfeebled brain. The tongue is clean—too clean; not red, but pallid. Its substance is œdamatous, and its edges indented. Such a person has no appetite, and the little he eats causes a weary sense of repletion until the ingesta slowly pass off. We may call this dyspepsia, as we may call the leg weakness dyskinesis, etc.; but the whole man is run down—call it general dysergy, if the phrase is acceptable. But now take another man equally without diathesis, and equally overworked; his tongue is bulky and also indented, and is protruding slowly, spreading as it issues. It is thickly coated, especially towards the mid-line, where the fur is brownish, and brownish towards the back also. The complexion is muddy, and the countenance bears the marks of mental depression. The hand is placed fretfully upon the vertex, where there is a pain, or if not a pain, a peculiar indescribable uneasiness; and this passes backwards surrounding the occiput. The urine deposits lithates, and the liver and stomach not only work feebly, but their functions are aberrant. He is weary and dull, and says he feels like a dead dog of a morning. A like state of tongue, and a like sluggishness and diversion of stomach, liver and colon, may be seen in most cases of common apoplexy, and are clearly secondary to the troubling of the brain, though the converse view is usually held, and the attack of apoplexy attributed to a foregoing upset of the digestion. Why, of the two overworked men, nervous exhaustion should produce in one a simply atony of the stomach, and in the other an aberrancy, I cannot say. I suspect the latter man has in himself some echo of gout, but yet I know how disappointing are all attempts to clean his tongue and set his stomach aright with the stock rhubarb and soda mixtures, with pepsines, with calomel, colocynth, or colchicum, and how that even strong tonics, such as quinine and iron, may be prescribed with benefit, and how rest and upland air will beat all medicines whatever, and will clean the tongue in a fortnight. Dyspepsia here is a symptom, and the stomach is disordered; but radically the state is a neurosis, vascular or other, and curable only upon this understanding.

That which I have illustrated by the examples of the stomach may be likewise illustrated by examples taken from the other viscera. Jaundice may be set up simply and directly by causes acting upon the nervous system, or it may be due to local causes only. Or again, it may owe its origin to some admixture of the two sets of causes. So again, in the functions of the intestines, either constipation on the one hand; or diarrhœa on the other, may be due to nervous causes acting alone, to local causes acting alone, or to local changes, reinforced by nervous reactions. I will only refer in this connection to one more organ out of many—to the uterus and its appendages. How intimately this organ, or this system, is associated with the nervous system, is well known, but, unfortunately, the weight of our knowledge all leans one way—it leans to a curious and busy search for every local ill which may arise in the female pelvis, while blind oblivion scatters the poppy over every outer evil, which in its turn might hurt the uterus—nay, more, a resolute prejudice would deny that, in the woman, any distress can arise which owes not its origin to these mischievous parts. "L'utérus c'est la femme," is a proverb which has received a new development in these days; for if by courtesy, rather than by conviction, woman be granted the possession of a few subsidiary organs, these, at best, have no prerogative nor any order of their own.

The uterus has its maladies of local causation, its maladies of nervous causation, and its maladies of mixed causation, as other organs have; and to assume, as is constantly assumed, that all uterine neuroses, or even all general neuroses in women, are due to coarse changes in the womb itself, is as dull as to suppose that the stomach can never be the seat of pain except it be the seat of some local affection, or that the face can never be the seat of tic-douloureaux unless there be decayed teeth in the jaw. All mucous membranes, indeed, seem readily to betray nervous suffering by relaxation or changed secretion; and I make no doubt whatever that a very large number of uterine disorders which are elevated to the place and name of diseases of the uterine system, are but manifestations of neurosis. All neuroses are commoner in women than in men. Facial neuralgia is commoner in them, migraine is commoner; so is gastralgia, again, and the pseudo-angina. Not only so, but, in the uterus, they possess one organ the more, with its own rich nervous connections, and its own chapter of added neuroses; but to say that all these maladies are due primarily to uterine vagaries, is to talk wide of all analogy. Again, some men as brave as others feel equal sums of pain far more acutely than the others; women, speaking generally, feel pain more than men do; patient as they are, they seem to have less reserve of force and less resistance, more susceptibility and resentment, and less capacity. Yet there is no standard of pain, nor of men, by which you shall say this patient is a coward, and his outcry exaggerated. Men and women are variously organized in respect of resistance to pain, and their fortitude or their despair must be tested, not by their cries, but by the other features of their characters. What right have we to say that a man writhing in the pangs of a toothache is a great sufferer, while, in the same breath, we hint that a woman complaining of pain in the abdomen is hysterical. The pain is equally invisible, equally unmeasured in the two cases, and the degree of credit to be given to the complaints is to be gauged by other probabilities.

A neuralgic woman seems thus to be peculiarly unfortunate. However bitter and repeated may be her visceral neuralgias, she is either told she is hysterical, or that it is all uterus. In the first case, she is comparatively fortunate, for she is only slighted; in the second case, she is entangled in the net of the gynæcologist, who finds her uterus, like her nose, is a little on one side; or again, like that organ, is running a little, or it is as flabby as her biceps, so that the unhappy viscus is impaled upon a stem, or perched upon a prop, or is painted with carbolic acid every week in the year, except during the long vacation when the gynæcologist is grouse-shooting, or salmon-catching, or leading the fashion in the Upper Engadine. Her mind, thus fastened to a more or less nasty mystery, becomes newly apprehensive and physically introspective, and the morbid chains are riveted more strongly than ever. Arraign the uterus, and you fix in the woman the arrow of hypochondria, it may be for life.

Now, gentlemen, it is time we complete our reaction from this gynæcological tyranny, and that we of this College no longer permit ourselves to be snubbed by these brethren of ours, who calmly tell us, with their superior airs, that our use of such expressions as uterine neuralgia, irritable uterus, ovarian neuralgia, neurasthenia, and the like, comes of a shallow sciolism, and is grounded upon the emptiness of our knowledge of uterine diagnosis.

The spirit of meekness alone restrains me from throwing the same stone again, and accusing our gynæcological friends of ignorance of neuropathies, and of the neurotic diathesis. That no man limited by the bounds of human intelligence can have a fine knowledge of medicine, surgery, and obstetrics is true, is as true of obstetricians as of physicians, but every man can have, and must have, a good all round knowledge of a kind which will enable him to take the main bearings of any case which may come to him.

For my own part, no conventionalities arrest me when my opinion is asked by a sufferer. The speculum and the uterine sound were invented as much for my benefit as for other people, aud I feel it both my duty and good will to examine into every detail of a case, whether medical, surgical, or pelvic.

This done, I am able to judge under whose care a patient may best be placed, and the more I thus incidentally learn of surgery and gynæcology, the more gladly and intelligently I recognize the extensive and dexterous attainments of those of my colleagues who work in those departments, and who can deal with such cases far better than I can. But, I repeat, and repeat again, that the main lines of the diagnosis of any malady whatsoever should be within the province and the abilities of any medical man whatsoever. What should we think of a family practitioner who made no diagnosis of a case of acute pneumonia, because his work lay chiefly with mothers, because he had not a stethoscope, and because he intended to call in a physician. On these grounds, I say that any well educated physician who does his duty to his cases, who does not idly turn them over to specialists, and who is armed with proper instruments of research, should know, as well as another, the main bearings of all his cases, and should claim to be heard on the diagnosis, although he may not and cannot pretend to distinguish minor differences, nor to have complete mastery of all the more refined devices of modern therapeutics. We physicians have been a feeble folk in this, we have shrugged our shoulders and submitted to gynæcological taunts in a way that may be very modest, but in a way that betrays our trust and our art. If the gynæcologists pelt us with stories of long pain and sickness uncured by medical futilities, but rapidly cured under uterine medication, we can mate their stories, and check them by double their number of cases received by the physician from the sofa, the manipulations and mental abasement of narrow uterine specialism. To underrate our debt to gynæcologists, to forget the great work they have done in the past half century, were as foolish as ungracious; but, like all great movements in special fields of inquiry, it must be subject to reaction, and its results must be checked by those which have been obtained by other methods and in other directions. The wisest and most disinterested of gynæcologists now know well how lamentable have been the exaggerations, how narrow the views, and how deceptive the data of many opinions which have passed current in their school, and they are ready to declare that if medicine is not wholly to reclaim a great part of the field occupied by them, its culture must at any rate be shared with the physician. The physician has been at least as much to blame, in that he has contemptuously thrown aside many cases of genuine malady and of genuine suffering as hysteria. Even hysteria is a complaint to be treated and relieved, but the central blunder has been the stupid confusion between the hysteric and the neurotic subject. On adding up the cases in my chamber note-book for 1883, I find that under the three heads of neuralgia, neurosis and neurasthenia, 151 new cases were entered in that one year. What a tale of misery does this limited experience of mine indicate! Add together all the patients of all the doctors in the West Riding alone and compute the manifold suffering. Yet, I do not hesitate to say, that in our vigorous northern people, hysteria is far from a like extension. On the contrary, if the word be used with due care, I would go so far as to say it is rare.

I remember but one case of hystero-epilepsy in the Leeds Infirmary in the last year or two, no cases of contracture, and of other hysterical affections but a few, and these often paraplegics, who relapse, and, returning twice or thrice to our care, swell the apparent numbers in our books. Take a hysterical person, man or woman, in its common and, so far, proper sense; take it to mean a person of feeble purpose, of limited reason, of foolish impulses, of wanton humors, of irregular or depraved appetites, of indefinite and inconsistent complaints, seeing things as they are not, often fat and lazy, always selfish; or, to take it in less degree, one capricious, listless, wistful, attractive perhaps, yet having always the chief note of hysteria—selfishness and feebleness of purpose; and if such persons complain of globus, of palpitation, which is never perceived by the stethoscope, of sleeplessness of which the nurse has no record, of dyspepsia which does not lessen the labors of the cook, of pains which never flush the cheek; and, if such person have, or have had, anæsthesia, unreal epilepsy, unreal syncope, unreal palsy, unreal cramps, then set down such person as hysterical, but forget not, nevertheless, to cure her mind and body. Such a patient is, no doubt, a member, a degenerate member, of the neurotic family; but it is almost with indignation that I repudiate the application of her adjective to the nervous sufferer, whom we may call the neuralgic member of that group. Why, gentlemen, my neurotic patients, if I can indicate them by a name, are almost the best people in this wicked world. Rarely endowed with the capacity and endurance, and profounder imagination, of the greatest, they form a large number of those in the second rank who are the salt of society. Let us suppose that Mr. Galton had photographed 400 of these, inside and outside; the resulting ideal neurotic might be much as follows:

His entry into your room tells of him at once. He enters with a brisk step, and a quick observant eye. You see a slightly built, meager man, of sallow complexion, or, if colored, with the color painted high upon the cheek-bone; the cheeks and the temples are hollow, the temporal arteries being visible under the lean skin, which often shows tanned markings, deepened during attacks of pain; the hair is straight, fine, and sparse upon the scalp; the features are sharp, often prominent, the lips thin and the skin dry; with it may be some remnants of eczema about the ears or chin. The tongue is protruded and retired quickly, and is generally narrow and pointed; it is rarely indented, and its tip, even when the health is best, does not cease to be red. There is often a slight silvery coating upon the dorsum and mid-line. The bodily frame is lightly and often finely built, the bony fingers and wrists and the visible sinews and radials betraying the absence of fat. Here and there, in later life, a knotty knuckle may tell of gouty parentage. The pulse, when most tranquil, usually ranges between 70 and 80, and accelerates on the least excitement. The clavicles and ribs, in like manner, are prominent, and the heart's apex may be seen to beat sharply before the eye. Its systole to the ear is likewise short and sharp, and the second sound very audible over a wide area. The limbs are small, but often very sinewy. Such persons are as active as birds, and the absence of fat in their muscles often gives to these, in states of health, the quality of hardness under the hand. Their conversation, again, is lively and voluble, often keen and brilliant, but impressionable rather than imaginative. Usually, such a patient does not readily come to you; he is brought, half reluctant, by his wife or a friend; he says, apologetically, he is an old dyspeptic, and you can do him no good. He has visited all the springs and half the doctors in Europe, and he lays a bundle of old prescriptions on your desk. Once agate, however, his story will be a long and minute one, but never maundering, wandering, nor whining. His companions will tell you that he is subject to great fluctuations of the animal spirits—gay, even fascinating, in society, brisk, orderly, and thorough in business, but at home dejected or fretful. He is a small eater, a light sleeper, and a worn worker. These persons are the heirs of every true neurosis, from insanity to toothache; and, on the whole, when we consider the infinite pertubations of intermarriage, it is surprising how true they run, or how clearly you may detect the neurotic strain in mixed descendants. Of their visceral neuroses, I shall have to speak hereafter, and would only say now that in both sexes of them migraine, stomach ache, and windy colic, are frequent and eminent, and receive the name of dyspepsia; and in the women are added to these uterine and ovarian neuralgias and hyperæsthesias. To call these suffering women of the neurotic type hysterical is to confuse all the due acceptance of names, and, what is worse still, it is to confuse the real relation of things. The neurotic woman is sensitive, zealous, managing, self-forgetful, wearing herself for others; the hysteric, whether languid or impulsive, is purposeless, introspective, and selfish. In the one is defect of endurance, but in the other defect of the higher gifts and dominion of mind.

Now, if we turn our eyes upon the flock of women who lie under the wand of the gynæcologist, we shall find it so largely composed of the neurotic and hysteric, that we may say in our haste the uterus has no substantial diseases ; that its affections are all neurotic, or so far reinforced by neurosis as to depend for their cure mainly upon neuropathic medicine. Herein we in our turn should be to blame. Many a woman, otherwise robust enough, and many a woman, whose weakness may lie not in her nervous system, suffers from uterine disorder, from painful uterine states, nay, even from distant sympathetic pains also, which come of mischief wholly local, or of mischief reinforced by diatheses other than the neurotic. Making, however, the utmost allowance for all these, I contend that a vast number, I will go further, and say a preponderating number, of such sufferers lie under the scourge of neurosis, and that their uterine and ovarian disorders are either wholly neurotic, or, as I have said, so reinforced by neurosis as to depend chiefly or wholly upon general medicine.

Let me take as an instance a young lady coming of a family in which great mental gifts had thrown into relief the many eccentricities and humours which accompany them ; a family, too, of which no household had been free from nervous disease. She possessed the gifts and the attractions of the neurotic diathesis, and labored under its defects. It is possible also that she was in some degree under the stress of what Anstie called the unconscious sexual impulse. She was restless, excitable, and suffering. Her pains were mostly pelvic and abdominal. She never put her feet to the ground, partly because it intensified her pain, partly because she had been forbidden to do so. She had lain on her back for months. Pessaries had been introduced, but, being intolerable to her, were withdrawn. Her periods were agonizingly painful for the first two days, and were profuse, and she had constant leucorrhœa. Her appetite was almost gone, her stomach queasy, her frame emaciated ; but she was unselfish and full of courage, and would have scorned the wiles and exacting whims of hysteria. Her womb had been incessantly under specular and other examination for a year or two, and, like nearly all such patients, she had uterus on the brain. I found the vagina tender, and the womb exquisitely tender; its substance was soft, and its attachments lax. Its position, therefore, was somewhat backward and downward. Acute suffering was caused in the upper hypogastrium when the fundus of the uterus was pressed upon *per rectum.* The rectum was full of fæces. By the speculum, I noted that there was both uterine and vaginal catarrh, and that the os uteri was excoriated—in the state, that is, of the upper lip of a scrofulous and snivelling little boy.

My most difficult task was to win my patient over to the belief that her disease was not mainly uterine, but mainly neuralgic; this once accomplished, our progress, though slow, was sure. I declined to initiate any treatment whatever until she would get her feet to the ground, and thenceforth cautiously regain the use of her legs. This took three months. Meanwhile I declined to "cure the ulceration of the womb" for the twentieth time, but made her content with rectal and vaginal astringent douches, first hot and afterward cold. As soon as she could walk, we perched her upon horseback. She was treated with the phosphide and valerianate of zinc, with bromide of ammonium, iron, quinine, and like remedies, with occasional sedative suppositories. In six months I found the uterus more compact, the ligaments braced, and the os clean and sound; the leucorrhœa had ceased, and all the parts could be handled without pain. Menstruation was still painful, but less so than formerly, and there was some menorrhagia. She was mixing, however, in general society, could ride gently to hounds, had regained appetite and looks; and, although I then lost sight of her, I have every reason to suppose she is as well as she is ever likely to become.

Now, gentlemen, is not this case one which in their degrees could be multiplied a hundredfold from our case-books or our memories; and yet these are they which form a great part of the women who are caged up in London back drawing-rooms, and visited almost daily for uterine disease, their brave and active spirits broken under a false belief in the presence of a secret and overmastering local malady, and the best years of their lives honored only by a distressful victory over pain.

The case I have described was selected by me because it was not one of mere irritable uterus without apparent disorder. Irritable uterus is a genuine malady, in spite of the denial given to it in high places. It corresponds to the hyperæsthesia of the stomach, which is found in the same diathesis, and which often simulates ulcer of the stomach.

But I pass over all these as neurotic enough, and I assert that, in such neurotic subjects, uterine laxities, moderate displacements, and catarrhs, owe their continuance, and often their very initiation, to an atonic state of body, and to a special instability of nerve-endowment, which may show itself in local trophic changes and in perverted secretions. Such changes or such settlements of perverted action are often, no doubt, called to this spot or the other by some local deviation from the normal, as a consumption may take its beginning from some trivial and forgotten catarrh; but the essence of the malady is not there, and to try to cure such a malady by local means is as wise as to try to cure a syphilis by antiseptic dressing of its ulcers.

Such subsidiary means are often needed—often, indeed, necessary; but in cases like those under discussion should be used as little as possible, because of the tendency of such methods to arouse and perpetuate a morbid possession of mind in the woman. All this our more robust, more clear-sighted, and more upright gynæcologists know well enough; in the rest, the fault may lie rather with modern fashion than with themselves.

Looking only to the uterine organs, their reason bounded by the confines of the pelvis, they attempt to stem the tides of general and diathetic maladies with little Partington-mops of cotton-wool on the ends of little sticks. That many of the cases we have discussed need a judicious combination of local with general treatment, is true, but in most of them the patient and the doctor are fascinated by the local phenomena, while nature herself is performing on a far larger scale. If we are to cure disease, we must be able to fly with her and to run with her, as well as to creep with her. In my later chapters I shall recall the truth which should be ever before us—that the fundamental difficulty in all neurotics, not hysterics, is their nutrition. More fresh air, without expenditure of the slender store of strength, the permeation of their starved tissues with the fat which they themselves so often loathe in their food—these two reforms accomplished, all their organs will take on a more generous and a more vigorous life, all their tissues will brace and cleanse themselves from a purer and richer fountain of blood, and force will be stored up and energy developed, wherein before was dilapidation and sterility. As a shrewd old Yorkshire doctor once said to me: "It's no use, my lad, putting the hands right upon the clock-face if you haven't cleaned the works."

Gentlemen, we are all one-sided; I speak to-day from my own one-sidedness, and my convictions are upon the side of cleaning and repairing the works. Sometimes, no doubt, when the general state of the health is restored, some local trouble set up originally by the constitutional state—be it gout, scrofula, or what not—smoulders on, forgotten, as it were, after the general malady is cured. For such local trouble diathetic treatment no longer avails; it must be wiped out by some local alterative. But, on the other hand, to contend daily with local troubles which daily are regenerated by some vicious habit of the whole system, is to roll up daily the shameless stone of Sisyphus.—*Brit. Med. Jour.*

